# Centralizing environmental datasets to support (inter)national chronic disease research: Values, challenges, and recommendations

**DOI:** 10.1097/EE9.0000000000000129

**Published:** 2021-01-25

**Authors:** Jeffrey R. Brook, Dany Doiron, Eleanor Setton, Jeroen Lakerveld

**Affiliations:** a Department of Chemical Engineering and Applied Chemistry, Southern Ontario Centre for Atmospheric Aerosol Research, University of Toronto, Toronto, Ontario, Canada; b Dalla Lana School of Public Health, University of Toronto, Toronto, Ontario, Canada; c Respiratory Epidemiology and Clinical Research Unit, Research Institute of the McGill University Health Centre, Montreal, Quebec, Canada; d Geography Department, University of Victoria, Victoria, British Columbia, Canada; e Department of Epidemiology and Data Science, Amsterdam Public Health Research Institute, Amsterdam UMC, VU University Amsterdam, Amsterdam, the Netherland; f Upstream Team, www.upstreamteam.nl, Amsterdam UMC, VU University Amsterdam, Amsterdam, the Netherlands.

**Keywords:** Environmental data, Environmental exposures, Data linkage, Harmonization, Data integration, Big data, Metadata

## Abstract

Whereas environmental data are increasingly available, it is often not clear how or if datasets are available for health research. Exposure metrics are typically developed for specific research initiatives using disparate exposure assessment methods and no mechanisms are put in place for centralizing, archiving, or distributing environmental datasets. In parallel, potentially vast amounts of environmental data are emerging due to new technologies such as high resolution imagery and machine learning.

**Objectives:**

The Canadian Urban Environmental Health Research Consortium (CANUE) and the Geoscience and Health Cohort Consortium (GECCO) provide a proof of concept that centralizing and disseminating environmental data for health research is valuable and can accelerate discovery. In this essay, we argue that more efficient use of exposure data for environmental epidemiological research over the next decade requires progress in four key areas: metadata and data access portals, linkage with health databases, harmonization of exposure measures and models over large areas, and leveraging “big data” streams for exposure characterization and evaluation of temporal changes.

**Discussion:**

Optimizing the use of existing environmental data and exploiting emerging data streams can provide unprecedented research opportunities in environmental epidemiology through a better characterization of individuals’ exposures and the ability to study the intersecting impacts of multiple environmental features or urban attributes across different populations around the world. Proper documentation, linkage, and dissemination of new and emerging exposure data leads to a better awareness of data availability, a reduction of duplication of effort and increases research output.

## Introduction

Environmental exposures and urban form are increasingly acknowledged to be important contributors to the development of noncommunicable diseases. Exposure to ambient air pollution has been recently recognized as a leading cause of global disease burden.^1^ Environmental attributes such as greenness, walkability, land use, noise, climate, and food environments are other established risk factors for chronic health conditions, as shown in different contexts around the world. Elucidating the relationships between such attributes and health outcomes and how they interact requires disentangling numerous correlated exposures characterized by small relative risks. Major challenges for environmental epidemiology in the coming decade are to channel information on environmental datasets available for research, ensure linkage to health datasets, standardize and sufficiently resolve environmental data across different populations, as well as make use of new data streams to characterize environmental exposures.

Research stakeholders, such as funding agencies in Europe^2^ and North America,^3,4^ are urging the research community to make a shift toward open science, data sharing, and collaborations as a means to advance scientific innovation and discovery. Regulatory agencies such as the European Commission and the Environmental Protection Agency (EPA) are also pushing for easier access to spatial and environmental data used in regulatory science decision-making.^5,6^ In parallel, potentially vast amounts of environmental data are emerging due to new technologies such as high-resolution imagery and machine learning. Such new data streams are offering unprecedented possibilities for environmental epidemiology by generating environmental datasets of high spatial and temporal resolution over larger and larger areas.^7^

To optimize the utility of existing and emerging environmental data for health research, structures and mechanisms should be put in place to ensure that data are findable, accessible, interoperable, and reusable (FAIR).^8^ In this essay, we argue that a more efficient use of exposure data for environmental epidemiological research over the next decade requires progress in four key areas: (1) establishing and promoting publicly accessible exposure metadata and data access portals, (2) facilitating and streamlining linkage with health databases, (3) adopting harmonized approaches to measuring and modeling environmental exposures over large areas, and (4) exploiting “big data” streams for exposure characterization and evaluation of temporal changes. A number of past and existing initiatives provide background and can support future developments in these areas.^9–13^

### Improving access to (meta)data and linkages with health data

Environmental epidemiologists often develop exposure metrics for specific research initiatives using disparate exposure assessment methods. Once developed, environmental exposure datasets are linked to health datasets which reside with individual researchers, and no mechanisms are typically put in place for centralizing, archiving, or widely sharing them. Whereas environmental data are increasingly available, it is often not clear how or if datasets are available for health research and metadata standards are lacking. The seemingly simple task of locating existing environmental exposure data available for research and understanding them (e.g., variable definitions, measurement methods, geolocation options) is in fact one of the most basic challenges faced by environmental health investigators.

Furthermore, considerable health data residing in the medical community are not applied in environmental epidemiology; either for lack of geographic identifiers at sufficient spatial resolution (e.g., home address or postal code) that are required to enable linkage with spatial environmental data, or due to the inability to send these identifiers to third parties for linkage due to privacy and confidentiality considerations. Given many health databases and cohorts *do* collect geographic identifiers, there are good and useful guidelines for doing so in a secure way within secure data facilities to protect privacy of individuals in administrative health databases or enrolled in observational cohorts. Since the majority of medical/health researchers are not equipped to generate their own state-of-the-art environmental data, efforts are needed to facilitate secure environmental and health data linkages.

Centralizing, documenting, linking, and disseminating environmental exposure datasets requires considerable resources and coordination. Organizations such as the Canadian Urban Environmental Health Research Consortium (CANUE)^14^ and the Geoscience and Health Cohort Consortium (GECCO)^15,16^ in the Netherlands are helping to fill these needs. Both infrastructures are academically funded and aim to collate and generate spatial measures of environmental exposures and urban form across Canada (CANUE) and the Netherlands (GECCO) in an effort to advance environmental health research. Environmental data housed in the CANUE and GECCO infrastructures are indexed to postal codes or small geographic areas such as those used in national Census, disseminated in simple, analysis ready formats via publicly accessible web portals and linked to health databases for broader distribution. Clear and detailed metadata of the available measures are provided, as well as technical information on procedures, operationalisations, and standards used to develop the data.^14,16^ To date, hundreds of research projects have been facilitated by data distributed via CANUE and GECCO. These projects, in turn, have furthered the evidence base and served as entry points for policy makers.^17–23^ In their respective countries, CANUE and GECCO are increasingly being recognized as a key source of environmental exposure data and facilitators of health and exposure data linkages through strong partnerships with administrative health data custodians and cohort studies.

### Standardizing new data for surveillance and epidemiological analyses

There is growing recognition among health researchers of the advantages of harmonizing and pooling health databases.^24–27^ These include increased statistical power to explore rare outcomes, small effects and interactions between risk factors, including gene-environment interactions, minimization of bias due to consistency in confounder adjustment and missing data, a better assessment of the robustness and generalizability of findings, and larger exposure ranges. Normalized difference vegetation index (NDVI), which estimates “greenness” or vegetation exposure from satellite imagery covering the entire planet is good example of a standardized metric used in epidemiological investigations around the world.^28^ The field of air pollution epidemiology has also spearheaded the use of standardized exposure data for cross-cohort and multinational collaborations. For example, the European Study of Cohorts for Air Pollution Effects (ESCAPE)^12^ and Effects of Low-Level Air Pollution: A Study in Europe (ELAPSE) projects^11^ have leveraged standardized approaches to measuring and modelling air pollution concentrations, and linked estimates to cohorts from across Europe to quantify and reduce the uncertainty of air pollutants’ health impacts.^29–33^ Globally standardized air pollution estimates combined with mortality rates and effect estimates from epidemiological studies have also allowed estimating the global burden of disease associated with air pollution exposure^1,34,35^ and has consequently helped drive public and policy awareness of the scale of impact of air pollution on human health. Nonetheless, relatively few environmental or urban exposures have been widely harmonized thus far and challenges remain. For example, few gold standards exist for environmental exposure assessment and the transferability of locally developed models is often limited. The importance of local context might also preclude the development of globally standardized metrics for certain environmental data (e.g., food environments, housing, walkability). Still, challenges to data standardization do not discount the potential benefits of developing and sharing approaches at some level of commonality. A balance might therefore be reached by developing less detailed but more consistent measures with a broader geographic coverage *as well as* more detailed measures that are better adapted to local context but cover smaller areas. For the latter, research continues to be needed to understand the geographic differences between the measures and how they may relate to health. Finally, new initiatives to expand the contents of global environmental datasets and to increase coverage in areas lacking data (e.g., low- and middle-income countries) can be expected to spark innovation in addressing these challenges or at the least better characterize when, where, why, and how context matters, helping environmental epidemiologists interpret findings and exploit geographic differences.

### Exploiting new data streams for exposure characterization

New data streams such as high-resolution satellite and street-level imagery combined with machine-learning techniques are providing, for the first time, local data for much of the urbanized world.^36^ For example, daily global satellite imagery is now available at 0.5 to 3 m spatial resolutions.^37,38^ Street-level imagery is also becoming ubiquitous, via proprietary sources such as Google Street View and openly via crowdsourcing efforts like Open Street Cam. Using these images, computer programs can be trained to identify urban features, which can be turned into geospatial data and used to estimate urban exposures appropriate for environmental health research. Machine-learning techniques and algorithms applied to satellite and street view images have been used to estimate air pollution,^39^ greenness,^40^ walkability,^41^ urban heat island intensity,^42^ and to predict spatial distribution of social and environmental health inequities.^43^ Ever-increasing coverage and resolution of these new technologies provide opportunities for building locally relevant but globally comparable environmental datasets across large geographical areas and can help bring data of equal quality to regions of the world where resources for environmental monitoring and surveillance infrastructure are limited.

### Recommendations

Optimizing the use of existing environmental data and exploiting emerging data streams can provide unprecedented research opportunities in environmental epidemiology through a better characterization of individuals’ exposures and the ability to study the intersecting impacts of multiple environmental features or urban attributes across different populations around the world. Key recommendations for a more efficient use of exposure data for environmental epidemiological research over the next decade are provided below.

First, national and international efforts should be directed toward collating and cataloguing existing and emerging datasets of area-level environmental exposures in central, publicly accessible web portals. Use of such web portals should be promoted in the research community and expansion of open data portals beyond national boundaries should be prioritized. Second, controlled vocabularies and compatible metadata standards should be developed and implemented for environmental exposure datasets. The use of compatible metadata standards across data platforms would facilitate multiplatform browsing and eventual data integration. Third, automated processes for indexing of spatial datasets to commonly used linkage fields such as points (e.g., addresses or postal codes) or small area census boundaries should be developed and implemented for existing and future spatial data streams. Fourth, systems and procedures to facilitate routine linkage of exposure files with health databases should be established. This requires substantial collaboration with health data custodians, potentially starting with existing international multicohort consortia, and with particular focus on addressing challenges presented by ethics, consent, and data confidentiality requirements. Fifth, once linked, health data holders should make both health and exposure data available via regular data access channels. Providing access to analysis-ready data will accelerate the research and discovery process. Sixth, and when possible, use of standardized measurement devices and modelling techniques should be prioritized for environmental exposure assessment to improve consistency of variables across studies. This includes exploring the potential of making use of historical exposure data covering large areas (national, continental) to generate compatible exposures. Seventh, international collaborations should be put in place to exploit opportunities offered by new technologies such as imagery and deep learning to scale up environmental exposures, with emphasis on the potential for exposure estimation in areas where lack of resources prevent environmental exposure monitoring and assessment. Finally, buy-in and ongoing support from funding agencies is needed to ensure sustainability and innovation in these areas.

## Conclusion

The CANUE and GECCO consortia provide proof of concept that centralizing and widely distributing environmental data for health research is valuable and can accelerate discovery in environmental epidemiology. While considerable investments are required for personnel (i.e., coordination, data scientists, and GIS specialists), data storage, and web development, these infrastructures have shown that proper documentation, linkage and dissemination of new, and emerging exposure data leads to a better awareness of data availability, a reduction of duplication of effort, and increases research output. Leveraging standardized exposures can also lead to larger sample sizes and the possibility of expanding research projects across different populations. We urge groups in other countries to set up open environmental data infrastructures in order to help catalyze novel research and collaborations on the environmental determinants of chronic diseases. The current COVID-19 pandemic has also revealed the relevance of health and environmental data linkages in infectious disease epidemiology.^23,44,45^

Ultimately, the environmental epidemiology and exposure sciences communities should work toward a global open data infrastructure capable of advancing knowledge on the health impacts of environmental exposures and informing policy for healthy city planning and hence, more-broadly, sustainable development. National and international science funding councils should allocate funds to support such initiatives in order to meet current and future data challenges and help advance the field of environmental health research.

## Conflict of interest statement

The authors declare that they have no conflicts of interest with regard to the content of this report.

## Acknowledgments

The Canadian Urban Environmental Health Research Consortium (CANUE) is financially supported by the Canadian Institutes for Health Research (CIHR). GECCO is financially supported by the Netherlands Organisation for Scientific Research (NWO), the Netherlands Organisation for Health Research and Development (ZonMw, 91118017), and Amsterdam UMC.
